# The role of prosody in interpreting causality in English discourse

**DOI:** 10.1371/journal.pone.0286003

**Published:** 2023-06-02

**Authors:** Na Hu, Aoju Chen, Hugo Quené, Ted J. M. Sanders

**Affiliations:** Department of Language, Literature and Communication, Institute for Language Sciences, Utrecht University, Utrecht, the Netherlands; Catholic University of the Sacred Heart: Universita Cattolica del Sacro Cuore, ITALY

## Abstract

Previous studies have well established that certain causal connectives encode information about the semantic-pragmatic distinction between different types of causal relations such as CAUSE-CONSEQUENCE versus CLAIM-ARGUMENT relations. These “specialized” causal connectives assist listeners in discerning different types of causality. Additionally, research has demonstrated that utterances expressing CLAIM-ARGUMENT relations exhibit distinct prosodic characteristics compared to utterances expressing CAUSE-CONSEQUENCE relations. However, it remains unknown whether the prosodic characteristics of utterances expressing causality can aid listeners in determining the specific type of causality being conveyed. To address this knowledge gap, this study investigates the impact of the prosody, specifically the prosody of the causal connective *so* in English, on listeners’ interpretation of the type of causality expressed. We conducted a perception experiment employing a forced-choice discourse completion task, where the participants were required to select a continuation for each sound clip they heard. The sound clip consisted of factual events followed by the causal connective *so*. We found that the odds of listeners choosing subjective continuations over objective continuations increased when the connective *so* at the end of the sound clip was pronounced with subjective causality prosodic features, such as prolonged duration and a concave f0 contour. This finding suggests that the prosody of the connective *so* plays a role in conveying subjectivity in causality, guiding listeners in interpreting causal relations. In addition, it is important to note that our data revealed individual variation among listeners in their interpretations of prosodic information related to subjective-objective causality contrast.

## Introduction

Establishing connections between discourse units is a prerequisite for understanding the structure of a discourse. According to Rhetorical Structure Theory [1], there are many different types of discourse relations (or, in the authors’ term, rhetorical relations) such as *concession*, *contrast*, and *cause*. Among these relations, *causality* is regarded as fundamental to human cognition [2, 3].

Based on discourse literature, human minds distinguish between at least two types of causality: subjective and objective [4–6]. This contrast is also termed as *pragmatic* versus *semantic* [3, 7], *internal* versus *external* [8], *diagnostic* versus *simple-causal* [9], and *presentational* versus *subject matter* causality [1]. The main difference between these two types of causality lies in how causality is constructed. Objective causality exists among events in the real world that are described by the speaker or author [10], such as the causal connection between an earthquake and collapsed buildings in example (1b). In contrast, subjective causality involves the speaker or author’s communicative acts or reasoning [11], as seen in (1a) where the conclusion “*The neighbors must be at home*” is inferred from the factual statement “*Their car is in the driveway*.*”* In such cases, the speaker or author is directly involved in the construction of the causal relation.

Therefore, a causal relation is subjective if it requires reference to the speaker, and objective if it does not. In other words, subjective utterances require an active *Subject of Consciousness* (SoC) for the interpretation [10, 12]. This *SoC* is the thinking entity in the discourse, who evaluates, as in the case of the subjective statement “*Utrecht is a lovely city*,” which involves the evaluation of the speaker as the *SoC*. Conversely, an objective statement such as “*Utrecht is a city in The Netherlands*” is presented as a fact in the physical world that does not depend on the evaluation of a *SoC*.

(1a) Their car is in the driveway. The neighbors must be at home.
(1b) There was an earthquake yesterday evening. Many buildings have collapsed.

The distinction between subjective and objective causality in human cognition is substantiated by evidence from language development and processing (see [13] for a detailed review). For example, research on child language acquisition has shown that the acquisition of these two types of causality takes place at different ages, with objective causality being acquired earlier than subjective causality [14, 15]. Furthermore, studies on language processing have found that these two types of causality have different processing patterns: subjective causality requires more cognitive resources to process than objective causality [9, 16, 17].

Numerous studies on discourse research indicate that these two types of causality can be distinguished at the morpho-syntactic level by coherence markers (also known as discourse markers or lexical markers of coherence and discourse structure; see [18]). For instance, subjective causality can be identified by cue phrases such as *I think* and *in my opinion*, which are suitable for (1a). In contrast, objective causality can be identified by phrases such as *for that reason* and *that’s why*, which are applicable to (1b) [19]. Apart from cue phrases, many languages around the world have different causal connectives to express subjective and objective causality, and language users systematically prefer one causal connective over another to convey a particular type of causal relation. For example, in Mandarin Chinese, the causal connectives *yushi* ‘so’ and *kejian* ‘so’ are exclusively used to express objective and subjective causality, respectively [20]. Other languages also have specialized causal connectives that indicate the intended type of causality, such as *daardoor* ‘so’ in Dutch [6], *parce que* ‘because’ in French [21], and *weil* ‘because’ in German [22], which are preferred for objective causality, while *dus* ‘so’ in Dutch, *car* and *puisque* ‘because’ in French, and *denn* ‘because’ in German are more commonly used for subjective causality (although the tendency is less robust than that observed with the objective causal connectives). These specialized causal connectives serve as processing instructions for causality, guiding the reader or listener in establishing the intended type of causality. For example, it is found in [23] that in Dutch, the subjective causal connective *want* ‘because’ causes a processing delay in the following words, while the objective causal connective *omdat* ‘because’ has no such effect. Similarly, it is found in [24] that in Mandarin Chinese, subjective (in the authors’ term, epistemic) causal relations marked with subjective causal connective *kejian* ‘so’ result in shorter reading time compared to those marked by an underspecified causal connective *suoyi* ‘so’. These findings suggest that subjective causal connectives such as *want* and *kejian* prompt the addressees to establish a subjective representation of the upcoming content. Furthermore, specialized causal connectives allow addressees to make predictions about the upcoming context. For example, research has shown that after hearing a subjective causal connective, addressees’ eye gaze fixates on an image depicting a person being interviewed by an interviewer [25], suggesting that the addressees expect the causality being communicated to involve a subject’s reasoning.

However, not all causal connectives can distinguish between subjective and objective causality. A typical example is the English connective *so* is a general causal connective that can express both subjective and objective causality [18, 26] and can fit both (1a) and (1b). Unlike specialized causal connectives such as *kejian* and *yushi* in Mandarin, general causal connectives, such as *so* in English, express a causal relation without specifying its type. As a result, readers can only determine which type of causality should be established by referring to contextual information, world knowledge, and other morpho-syntactic cues.

In speech communication, information is conveyed not only through morpho-syntactic means that involve choice of words and syntactic constructions but also through other means. As one of the non-lexical means of communication, prosody, referring to the acoustic variations in speech [27], carries a variety of meanings and functions in speech (for extensive reviews, see [28–31]). For example, prosody signals syntactic structures (e.g., [32]), marks discourse and rhetorical structure (e.g., [33–36]), marks focus and information status [37–39], conveys pragmatic meanings such as uncertainty, irony, and sarcasm (e.g., [40, 41]; see [42] for an extensive review), helps interlocutors with conversational turn-taking [43], and reveals speakers’ social-indexical identities such as region, gender, and ethnicity. Furthermore, research also shows that prosodic information contributes to speech processing (see [28, 30] for reviews). For example, prosody has been found to help listeners resolve syntactic ambiguity (e.g., [32, 44]), interpret focus or information status (e.g., [45]), and resolve referential ambiguity (e.g., [46–50]), establish the structure of a discourse [51], grasp illocutionary force and affective meaning (e.g., impoliteness: [52]; sarcasm: [40]), take turns in dialogues, and detect the function of polyfunctional words or word combinations such as *yes* and *I think* in a discourse [53, 54].

Regarding the role of prosody in expressing different types of causality, a previous study has shown that prosody plays an active role in expressing subjective and objective causality in English in the absence of morpho-syntactic cues for these two types of causality [55]. Specifically, subjective causality in forward order (ARGUMENT-CLAIM) expressed by the general causal connective *so* has a higher pitch in the second clause (the clause that contains a claim), a lengthened *so*, and a unique f0 contour for *so*, compared to objective causality (CAUSE-CONSEQUENCE). Based on this finding, the current study sets out to investigate whether listeners can distinguish between subjective and objective causality based solely on the prosodic differences in the absence of morpho-syntactic markers.

Previous research on the use of prosody to process information has also found that, while at the group level listeners make use of prosodic information to understand utterances, there exists substantial variation at the individual level, with some listeners being more successful than others in decoding the information conveyed by prosody. This individual variability has been reported in the perception of prosodic information associated with various aspects, such as information structure [56], including focus types [57] and prosodic phrase boundaries [58]. Furthermore, it has been noted in the interpretation of irony [59] and sarcasm [60]. In the current study, we aimed to explore whether listeners differ in their interpretation of prosodic information related to the contrast between subjective and objective causality.

In short, the aim of this study is to investigate whether prosody has an effect on the processing of subjective and objective causality in English when the type of causality remains unspecified at the morpho-syntactic level. This is of particular interest because the English general causal connective *so* does not specify the type of causality intended by the speaker. Additionally, the study aims to explore whether the effect of prosody differs among listeners. We conducted a perception experiment online, using an forced-choice discourse completion task similar to the task employed by [53]. In our task, the participants heard short audio clips narrating real-world events ending with the connective *so* (e.g., “*Jim got his nose pierced so”*), with *so* being realized in two different types of prosody: one associated with utterances conveying subjective causality (henceforth referred to as “subjective causality prosody”), and the other associated with utterances conveying objective causality (henceforth “objective causality prosody”), as documented in previous literature. After listening to each stimulus, the participants were tasked with choosing one continuation from the two options provided on the screen for the stimulus they heard. One continuation was an opinion or inference about the event described in the stimulus (hereafter referred to as “subjective continuation”), forming a subjective causal relationship with the stimulus; the other was the consequence of the event stated in the stimulus (hereafter “objective continuation”), thus forming an objective causal relationship with the stimulus. We predicted that listeners will opt for subjective continuations more often when the causal connective *so* is pronounced with subjective causality prosody (i.e., longer duration and a concave f0 contour) than when it is pronounced with objective causality prosody. However, as the perception and interpretation of prosodic information often varies among individuals [56, 57], we anticipate that the effects of the prosody of the connective *so* might differ across participants.

## Method

### Participants

A total of 55 participants (27 females and 28 males) participated in the experiment. The participants were recruited from Prolific (www.prolific.co), an online crowdsourcing platform. A set of screening criteria was set up on Prolific to ensure the smooth running of the experiment. To be eligible for the study, participants had to be native speakers of American English, between the ages of 25–35, raised in a monolingual family, and had obtained a bachelor’s degree. The participants received a small participation fee.

### Materials

The materials used in this experiment were adopted from [55], which explored the acoustic differences between utterances expressing subjective and objective forward causality in the absence of specialized causal connectives. The study included 15 pairs of target items, each consisting of two items, one expressing objective causality and the other expressing subjective causality (see (2a) and (2b)). The first clause (shown in the square brackets with the subscript “event” in (2a) and (2b)) was identical for each pair of items, telling a real-world event (hereafter referred to as the “event” clause), such as “*Jim got his nose pierced”* in (2a) and (2b). In the present experiment, the 15 “event” clauses were presented to the participants in an online forced-choice discourse completion task (as described in the “Task” section) in audio form, followed by the connective *so* (e.g., “*Jim got his nose pierced so”*), with the connective *so* having two different types of prosodic realizations (subjective versus objective causality prosody), resulting in a total of 30 audio stimuli. While the first clause was identical for each pair, the second clauses were different, shown in square brackets with the subscript “continuation” in (2a) and (2b). For the objective item in each pair, the second clause stated an actual consequence of the event described in the first clause, e.g., “*He bled a little”* as in (2a), thus forming objective forward causality with the first clause. For the subjective item in the same pair, the second clause expressed an opinion or evaluation of the event stated in the first clause, e.g., “*He wants attention”* as in (2b), thus establishing subjective forward causality with the first clause. In the present experiment, these two clauses were presented to the participants on the screen as two optional continuations to the audio stimulus they heard. In addition to the target items, 20 fillers were adopted from [55] to conceal the research goal from the participants. All filler items expressed concessive relations with *but* (see (3)) and had a sentence structure similar to the target items.

(2a) [Jim got his nose pierced] _event_ so [he bled a little] _continuation1_.
(2b) [Jim got his nose pierced]_event_ so [he wants attention] _continuation2_.
(3) [John worked very hard] _event_ but [he failed his exams].

### Speech stimuli

The audio stimuli used in the present experiment consisted of 30 recordings (15 tokens two prosodic versions). These recordings were created based on utterances collected by [55]. The utterances were suitable as stimuli for the current study because they were produced by speakers a naturalistic conversation setting in which they responded to questions posed by an interlocutor. The study had involved 15 native speakers of American English. To select the speaker for the current study, four judges independently evaluated the speech samples produced by each speaker in terms of voice quality (creakiness), fluency (pauses within a clause), and articulation rate (number of syllables produced per second). The ideal speaker for the current study should have a non-creaky voice, speak fluently, and maintain a moderate speaking rate. The speaker performed best in all of these respects, as unanimously selected by the judges, is a male speaker aged 23 (at the time of participation), and his utterances were used to create the stimuli for the current study.

To ensure that the prosody of each utterance produced by this speaker matched the type of causality expressed in the utterance, a series of acoustic manipulations were applied to the utterances. The manipulations were based completely on the findings of [55] and involved only the prosody of the connective *so*, not the prosody of the “event” clause, because it was found in [55] that the prosodic differences between subjective and objective causality lie in the connective *so* and the second clause (not involved in the current experiment), not in the first clause (the “event” clause). Specifically, it was reported in [55] that the connective *so* had a longer duration (estimated mean difference: 50 milliseconds (ms), 95% CrI (95% credible interval, the central portion of the posterior distribution that contained 95% of the plausible values) [30 ms, 70 ms]) when expressing subjective causality than when expressing objective causality. Based on the estimated mean difference and intercept reported in [55], we derived a mean duration of 285 ms for the connective *so* in the subjective condition and 235 ms in the objective condition. In addition to the durational difference, [55] also reported a difference in the f0 contour for the connective *so* between subjective and objective causality. In [55], the f0 contours of the connective *so* had been modeled using Functional Principal Component Analysis (FPCA) [61]. This method converts each contour into a mathematical function of time (see Eq 1), which consists of the average contour of all contours in a data set (*μ(t)*) and three Principal Component contours (*FPC1(t)*, *FPC2(t)*, and *FPC3(t)*), each FPC being associated with a weight (*s1*, *s2*, and *s3*) that indicated the extent to which each FPC should be applied to the mean contour in order to reconstruct the original contour with a reasonable balance between overfitting and under-fitting.


F0(t)≈μ(t)+s1∙FPC1(t)+s2∙FPC2(t)+s3∙FPC3(t) (1)

**Fig 1:**
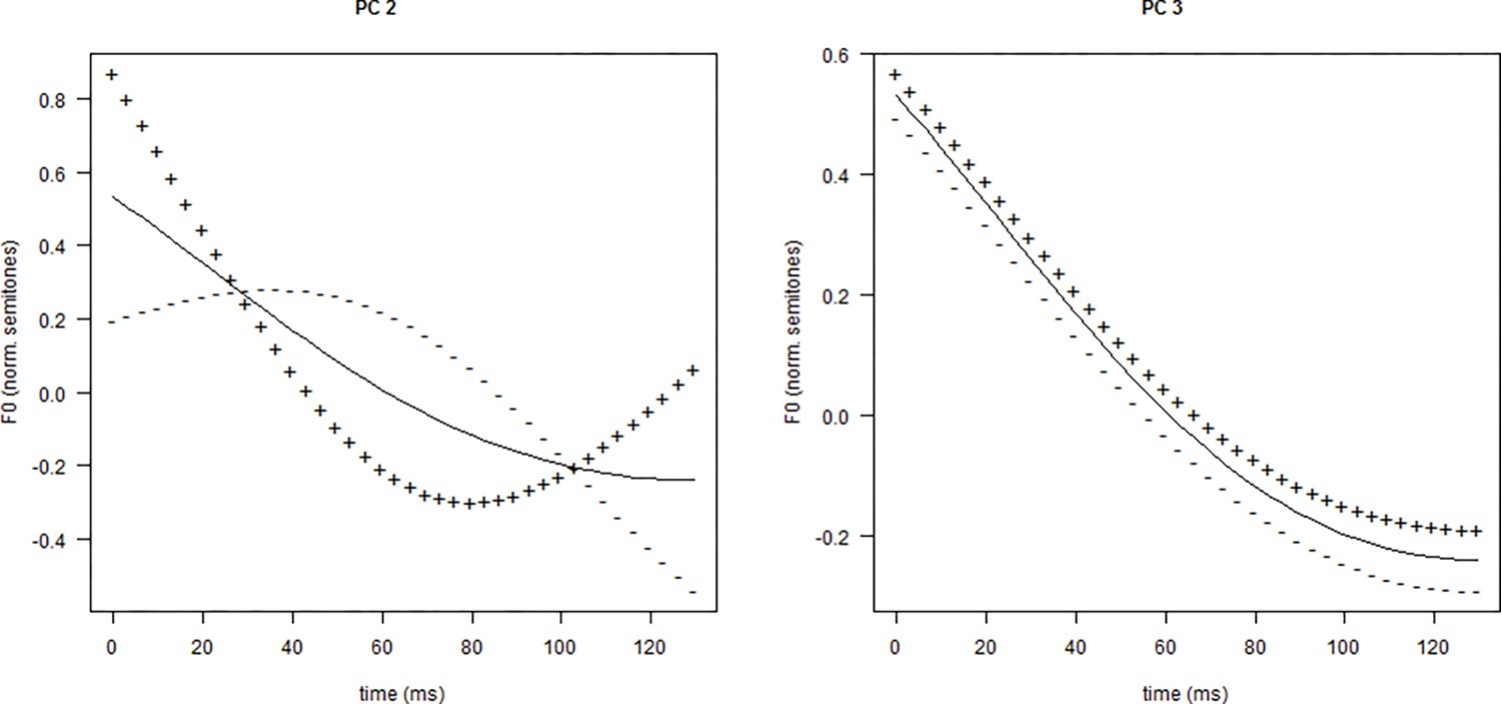
The effect of FPC2 and FPC3 on the mean f0 contour of the connective (left: FPC2; right: FPC3). The solid line in each panel represents the mean f0 contour (normalized f0 in semitones over time in ms); the “+” line illustrates the outcome curve after adding one standard deviation of the FPC to the mean f0 curve; the “-” line shows the outcome curve after subtracting one standard deviation of the FPC from the mean f0 curve. The x-axis denotes time in ms, and the y-axis represents normalized f0 in semitones.

It was found in [55] that the weight of FPC2 was 0.85 units larger (95% CrI [0.24, 1.46]) and the weight of FPC3 was 0.09 units smaller (95% CrI [−0.19, 0.00] for contours in the subjective condition than those in the objective condition. The impact of these two FPCs on the mean f0 contour of the connective was shown in Fig 1. In descriptive terms, these estimates suggest that the f0 contour of the connective *so* was more concave and had a lower intercept in subjective causality than in objective causality.

**Fig 2:**
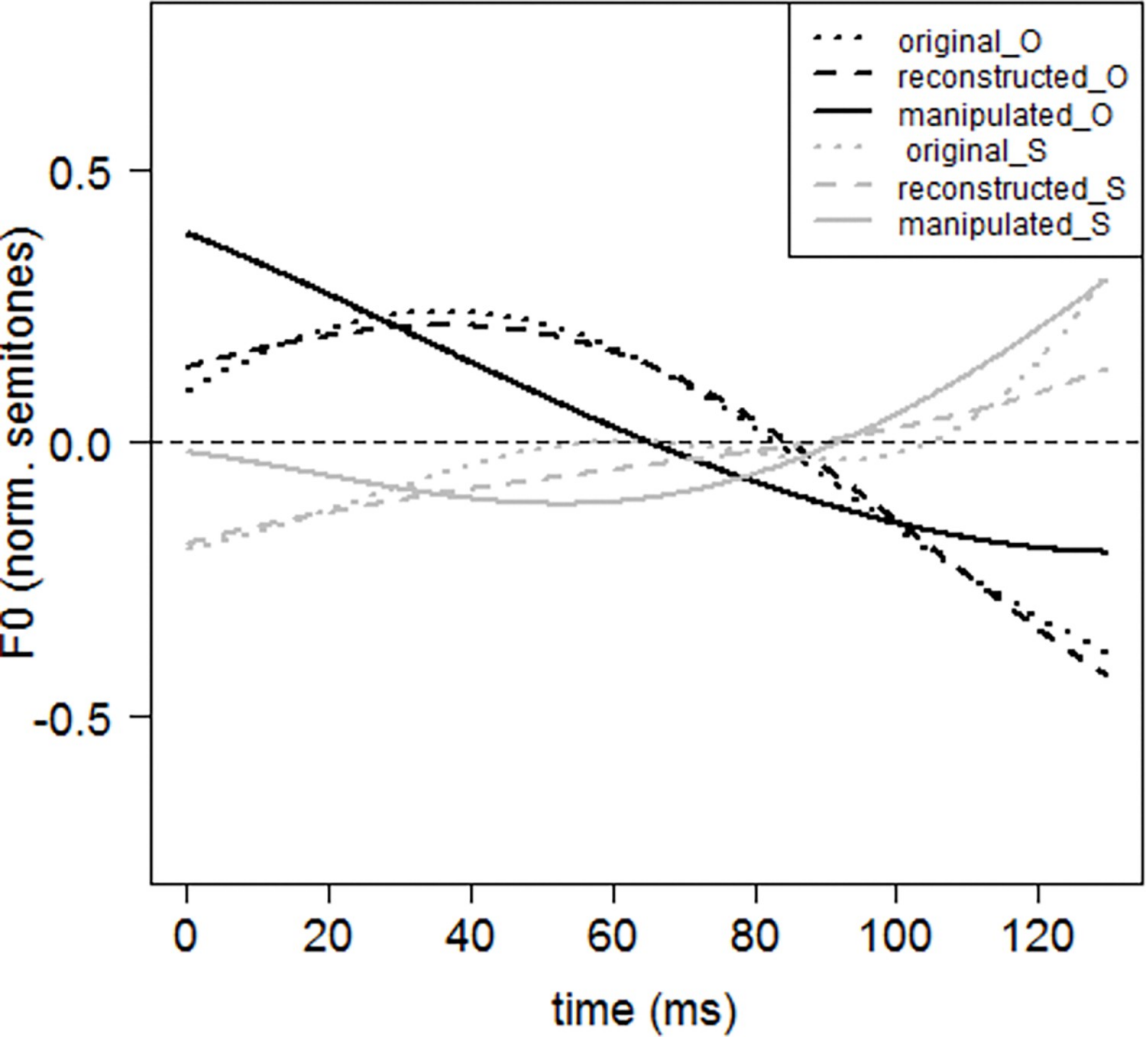
The f0 contours of the vowel /əʊ/ in the connective *so* for the two stimuli in pair 5. The lines are color-coded as black for the objective causality prosody condition (“_O” in the legend) and grey for the subjective causality prosody condition (“_S” in the legend). The dotted lines show the original f0 contours (“original”); the dashed lines represent the resulting f0 contours after FPCA (“reconstructed”); the solid lines display the resulting f0 contours after manipulation, where the values of FPC2 and FPC3 in Eq 1 were changed to the mean values estimated by the linear regression (“manipulated”).

Based on the model estimations reported in [55], we derived that the mean weight of FPC2 was +0.335 units for subjective contours and −0.515 units for objective contours, and the mean weight of FPC3 was −0.065 units for subjective contours and +0.025 units for objective contours. These values were used to create a typical contour for the connective *so* for each type of causality (see Fig 2).

The acoustic manipulations on the recordings of the utterances were carried out by changing the duration and f0 contours of the connective *so* using Praat [62] (for data extraction, duration manipulation, and speech synthesis) and R [63] (for f0 contour restyling using FPCA (code is available at https://osf.io/g8e6x/?view_only=ca49622082d84eb4a09830105e1b2fce). The process involved eight steps:

1. The first clause (the “event” clause) and the subsequent connective *so* were extracted from each utterance as a new audio file, resulting in 30 sound clips.
2. The f0 contours of the connective *so* (dotted lines in Fig 2) were modelled using FPCA with duration normalized, resulting in 30 mathematical functions in the form of Eq 1 (dashed lines in Fig 2).
3. The weights of FPC2 and FPC3, i.e., s2 and s3, in each obtained function were modified based on causality conditions (solid lines in Fig 2).
4. The value of F0(t) in each obtained function was solved for every 5 ms, yielding a new series of f0 values, i.e., a new f0 contour for the connective *so*.
5. The new f0 contours were used to replace the original contours in the clips, which were then resynthesized into speech using the PSOLA synthesizer in Praat.
6. The duration of the connective *so* in each synthesized audio was modified based on causality conditions. To avoid disproportional changes in the duration of the two phonemes, the original ratio of segment duration was preserved.
7. The silent interval between the “event” clause and the connective *so* in each stimulus was removed to exclude its potential effect on the interpretation of subjective and objective causality.
8. The authors evaluated the naturalness of the utterances perceptually, and the evaluation showed that all stimuli were natural in terms of prosody. Table 1 summarizes the acoustic specifications of the two resulting types of prosody.

**Table 1:** The acoustic details of the connective so in each prosodic condition.

Prosodic version	The shape of the F0 contour for the connective	Duration of the connective
subjective	μ(t) + s1 * PC1 + (+0.335) * PC2+ (−0.065) * PC3	285 ms
objective	μ(t) + s1 * PC1 + (−0.515) * PC2+ (+0.025) * PC3	235 ms

Filler stimuli were prepared based on the speech samples produced by the same speaker. For each utterance produced by the speaker, the part from the beginning of the first clause till the end of the connective *but* (e.g., “*John worked very hard but”* in (3)) was extracted using Praat, resulting in 20 filler stimuli. No acoustic manipulation was performed on the filler stimuli.

### Task

**Fig 3:**
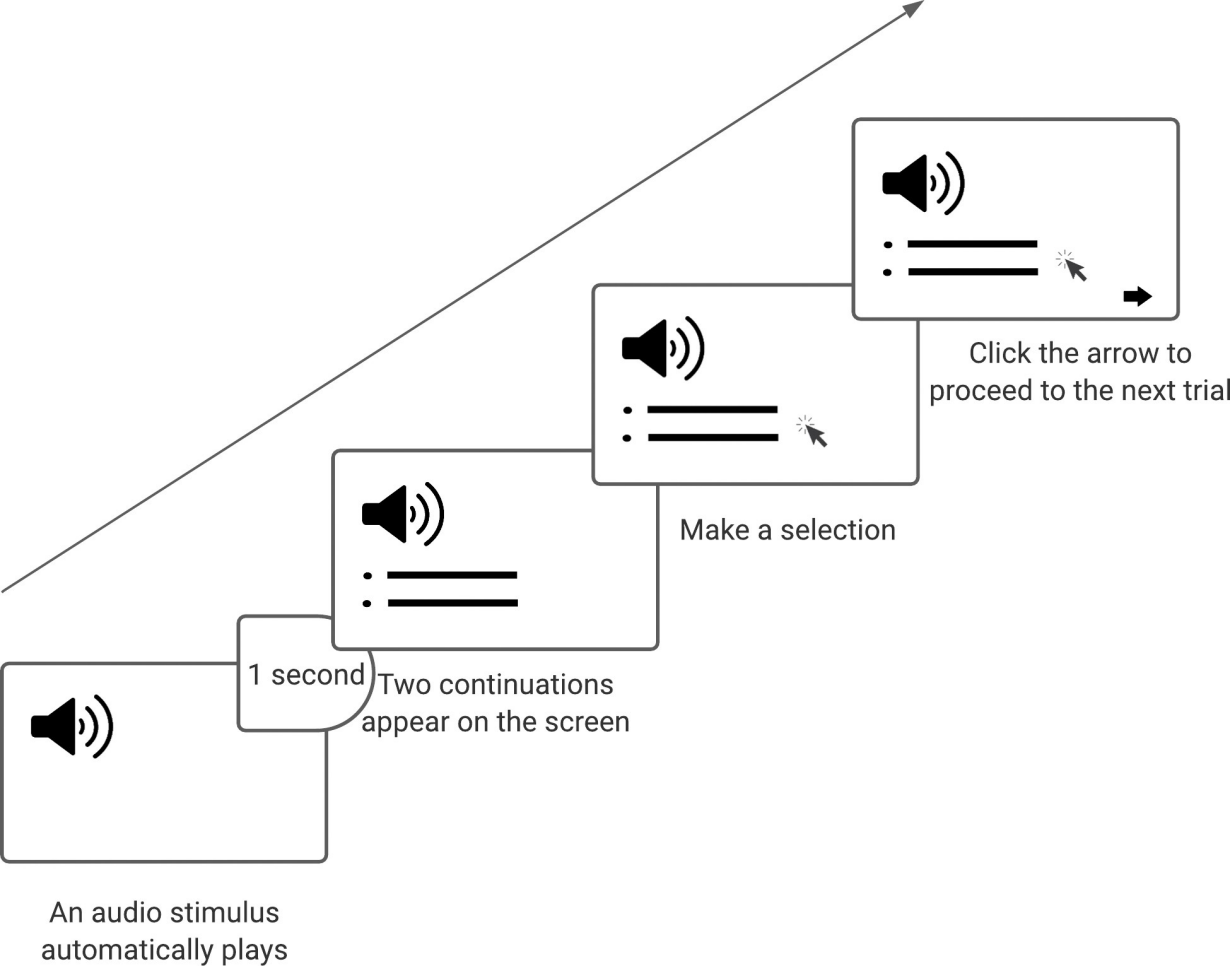
A graphic description of the timeline of a trial.

The stimuli (comprising 30 targets and 20 fillers) were presented to the participants using an online forced-choice discourse completion task set up with the online survey tool Qualtrics (www.qualtrics.com/). During the task, the stimuli were presented to the participants in a randomized order, one trial at a time, over headphones. On each target trial (trials involving target stimuli), a stimulus, e.g., “*Jim got his nose pierced so”*, was first played automatically to the participants. One second after the stimulus was played, two possible continuations of the stimulus appeared on the screen in text format, one unveiling the consequence (e.g., “*He bled a little”*) of the event announced in the stimulus, the other expressing an opinion/deduction/inference (e.g., “*He wants attention”*) about the event (see Fig 3 for a graphic description of the trial timeline). The participants’ task was to choose the continuation that they thought best continued the stimulus they had just heard. Throughout the task, the play button remained visible on the screen, allowing participants to replay the stimulus as many times as they wanted before making their choice.

The filler stimuli were presented to the participants in the same way as the target stimuli. On each filler trial, a filler stimulus (e.g., “*John worked very hard but”*) was played first. Then two continuations were shown on the screen, of which only one satisfied the concessive relation required by the connective *but*, and the other sentence had no semantic relevance to the stimulus. Take the stimulus “*John worked very hard but”* as an example. The two continuations shown on the screen were (A) “*He failed his exams”* and (B) “*The plane landed on time*.” Option A was the only correct choice, while Option B was completely unrelated to the context. In the experiment, the order of the correct and incorrect options was randomized. Considering that the correct continuation was evident, the filler stimuli were also used as attention checks. Participants who selected incorrect continuations in 10% or more of the filler stimuli were judged as having failed the task, and their responses were excluded from further analyses.

### Procedure

The experiment had been approved by the Ethics assessment committee of Humanities at Utrecht University prior to its implementation. The experiment was conducted online via the crowdsourcing platform Prolific (www.prolific.co). Prolific users who met the screening criteria (refer to the *Participants* section for details) received an invitation via email. Users who accepted the invitation first completed a Captcha test, which was used to filter out bot users (as identified by Prolific). Those who passed the Captcha test read an information letter on the screen that provided relevant information about the study including the nature of the task, data storage, and approval policies. After reading the letter, users had the opportunity to decide whether they wanted to participate in the experiment. Users who did not wish to proceed could simply click “No” on the screen to withdraw from the survey. The users who agreed to participate were asked to provide their Prolific ID (which served as the unique identifier for their submissions and subsequent communication regarding the study’s outcome). The participants then read instructions (see S1 Appendix), which explained the task and instructed them to wear headphones to listen to stimuli and complete the task in a quiet room. After that, the survey started. The entire experiment took approximately 15 minutes to complete. The participants were remunerated 2.5 euros for their participation through Prolific.

### Statistical analysis

We first examined the participants’ responses to the filler questions. We found that out of the 55 participants who submitted their responses, 38 participants answered the filler questions with 100% accuracy, 2 participants achieved an accuracy rate higher than 90%, 3 participants had an accuracy rate between 70% and 89%, 8 participants had an accuracy rate between 50% and 69%, and 4 participants had an accuracy rate lower than 50%. Based on the predetermined assessment criteria, we excluded participants with accuracy rates below 90% from further analyses. Consequently, the final sample consisted of 40 participants.

To estimate the effect of the prosody of the connective *so* on the participants’ choice of continuation, we fitted a series of Bayesian logistic regression models in R (version 3.6.3, R Core Team, 2020) [63] using the brms package [64], a wrapper package for the probabilistic programming language Stan [65]. The outcome variable in our models was a dichotomous variable indicating participants’ choice of continuation (subjective continuation = 1, objective continuation = 0). The predictor variable was the prosody of the connective *so* (prosody), which had two levels: subjective and objective, coded as 1 and 0, respectively. The grouping variables were participant and pair (verbally identical but acoustically different stimuli were considered a pair). We added these variables into models in an incremental manner (see Table 2), starting with an intercept model (m0) containing only the grouping variables participant and pair. Subsequently, we constructed m1, which contained the predictor variable prosody, followed by m2, which incorporated a varying slope for the effect of prosody by participant (1+prosody|participant), allowing for by-participant adjustments to the effect of prosody. Lastly, we constructed m3, which involved a varying slope for prosody by pair (1+prosody|pair), allowing for by-pair adjustments to the effect of prosody.

**Table 2:** Model formulas.

Model	Formula
m0	~ 1 + (1|participant) + (1|pair)
m1	~ prosody + (1|participant) + (1|pair)
m2	~ prosody + (1+prosody|participant) + (1|pair)
m3	~ prosody + (1+prosody |participant) + (1+prosody|pair)

To evaluate each model term, we computed the Bayes factors for each model term using the function bayes_factor (m1, m0) in brms, where m1 and m0 in the bracket refer to models with and without the model term being evaluated, respectively. For example, to assess the effect of prosody on the participants’ choice of continuation, we compared m1 and m0 in Table 2, which were the models with and without the constant effect term prosody, respectively. The resulting Bayes factors, referred to as *BF*_*10*_ (with the subscripts 1 and 0 referring to m1 and m0, respectively), indicate the extent to which the evidence supports m1 over m0, akin to the ratio of likelihoods of the two models [66, 67]. A larger *BF*_*10*_ value indicates stronger support for m1. The conventional interpretations of *BF*_*10*_ are as follows: *BF*_*10*_ greater than 10, 3–10, and 1–3, respectively, indicate strong, weak, and very weak support for m1; *BF*_*10*_ smaller than 0.1 indicates that the evidence favors m0 [68].

**Fig 4:**
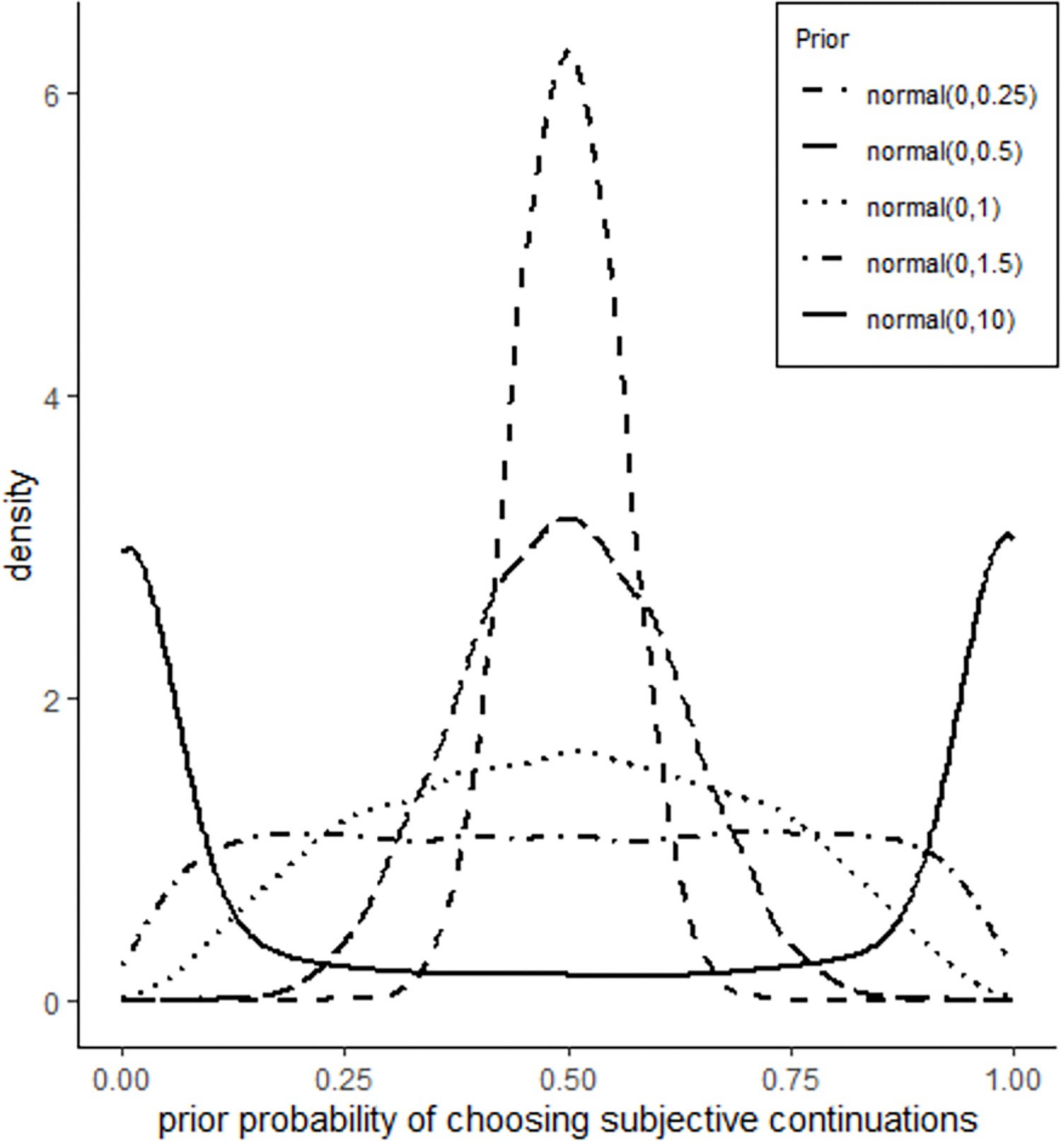
Prior predictive distributions generated by m0, the intercept model, under different priors.

It is worth noting that Bayes factors can be affected by the prior assumptions incorporated in models [69], that is, the assumption held by the model before the data comes in. As a result, interpreting *BF*_*10*_ may be challenging To circumvent this issue, it is common practice to compute Bayes factors under different priors [69]. In our study, we took this approach and calculated Bayes factors for each effect of interest using three different priors. We selected normal distributions (normal (0, SD)) with standard deviations (SD) of 1.5, 1, and 0.5 for the parameters of interest in the model. These priors were chosen after examining the prior predictive distributions generated by the intercept model incorporating priors (normal (0, SD) with SD of 10, 1.5, 1, 0.5, and 0.25. The results of the prior predictive checks showed that models incorporating priors with SDs of 1.5, 1, and 0.5 produced reasonable prior predictive distributions (shown in Fig 4). In descriptive terms, the models did not have strong beliefs about which continuation the participants would choose before seeing the actual data. In contrast, the model incorporating the normal (0, 10) prior, a conventional flat prior commonly used in linear regression models, produced highly unrealistic data. This model assumed that the participants either never or always chose the subjective continuation. Since this was a very extreme assumption, the normal (0, 10) prior was not adopted. The reason that the normal (0, 10) prior does not behave as a flat prior in the current case as it does in linear regression models is that the parameter values in logistic models are in the log-odds space, which are hard to interpret. To achieve interpretability, it is necessary to convert these parameter values to the outcome probability space using an inverse-link function (in this case, the function is inv-logit, as the link function we used is logit) [70]. The resulting values tend to concentrate near zero and one, as shown in Fig 4. The models were fitted using four chains, 10,000 iterations, of which 2,000 were in the warming up phase, and the Bernoulli family with the logit link function, which maps a parameter that is defined as a probability mass onto a linear model that can take on any real value. To assess model convergence, we verified that there were no divergent transitions and the Rhat value for each model coefficient was close to one. We also ensured that the number of ESS (effective sample size) exceeded 90% of the post-warmup samples, following [71].

As previously stated, the model parameters for logistic models are expressed in the log-odds space, making them difficult to interpret. Specifically, the coefficient associated with a predictor variable represents the change in log-odds of the response variable for a one-unit increase in the predictor variable. To make this model parameter more easily interpretable, it was exponentiated using the function exp(). The resulting value represents the ratio of the odds of choosing subjective continuations after hearing stimuli with subjective causality prosody to the odds of choosing subjective continuations after hearing stimuli with objective causality prosody.

In the following result sections, we first focus on the intercept of m0, which represents the baseline of the participants’ choice of continuation. Then, we present the results from m3, which has the most complete random components. The summaries of m1 and m2 are provided in the supplementary material. For each model parameter of interest, we report the estimated mean and the 95% credible interval (95% CrI, representing the interval between the 2.5th and 97.5th percentiles of the posterior distribution). Additionally, we report the Bayes factors to assess the likelihood of each effect’s presence in our data, along with the priors employed for computing the Bayes factors. Unless otherwise stated, the posterior distributions reported in the main text were all estimated by the model with the least informative prior normal (0, 1.5).

## Results

First, the intercept model (m0) showed that the posterior distribution of the intercept was centered at −1.06 with a 95% CrI [−1.34, −0.77]. Considering that the estimated 95% CrI did not include zero, we can be certain that the estimated mean was not zero. Since this model did not contain any predictor variables, the intercept of the model represented the baseline log odds of the participants choosing subjective continuations. By exponentiating the log odds, we obtained the odds of participants choosing subjective continuations, which is 0.34 with a 95% CrI [0.26, 0.46]. With this information, we further derived the odds of participants choosing objective continuations, which is 2.94 (1/0.34), higher than the odds of choosing subjective continuations. This suggests that the participants had a strong baseline tendency to choose objective continuations for the stimuli they heard.

**Fig 5:**
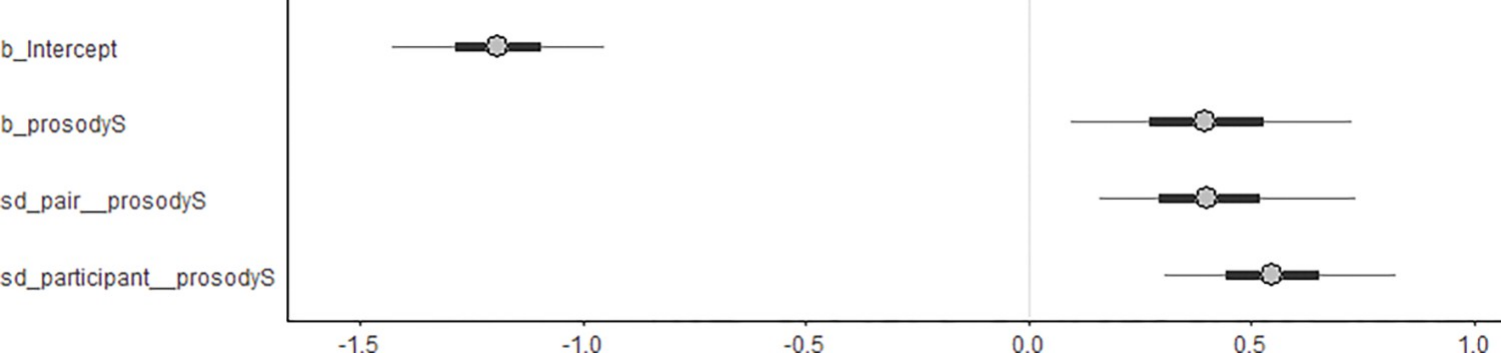
The posterior distributions of model parameters of the most complex Bayesian mixed-effects model (m3) with the least informative prior for the constant effect of prosody. The model contains the constant effect of prosody, which consists of two levels: subjective and objective (coded as 1 and 0, respectively). Additionally, the model incorporates a varying slope of prosody by participant and pair. The plot shows the estimated mean (as represented by circles) as well as the lower and upper bounds of the 95% (depicted by thinner lines) and 50% (thicker lines) credibility intervals for each model parameter of interest. These parameters include the random effect terms sd(prosodyS) by participant and pair, the intercept, and the constant effect of prosody.

Fig 5 illustrates the estimates of the most complex model—the model that contained the constant effect prosody and by-participant and by-pair random slopes of prosody (m3 in Table 2). According to the model, the coefficient associated with prosody is estimated at 0.39 (95% CrI [0.03, 0.76]). Table 3 shows the Bayes factors for the constant effect prosody, which ranged from 3 to 7, showing weak support for this effect. The estimated mean for this coefficient indicates that the log-odds of selecting subjective continuations will increase by 0.39 units for every unit of change in prosody, specifically from objective to subjective causality prosody. After exponentiating these estimates, we found an odds ratio of 1.47 (95% CrI [1.03, 2.14]) for the prosody effect, indicating that across participants and pairs, the odds of choosing subjective continuations in the subjective causality prosody condition were 1.47 times the odds of choosing subjective continuations in the objective causality prosody condition.

**Table 3:** The Bayes factors for each model term, computed using three different priors.

parameter	Prior	BF10
~Pair sd(prosody)	Normal(0, 1.5)	5.34
	Normal(0, 1)	7.60
	Normal(0, 0.5)	11.4
~Participant sd(prosody)	Normal(0, 1.5)	32.7
	Normal(0, 1)	45.1
	Normal(0, 0.5)	61.7
prosody	Normal(0, 1.5)	3.02
	Normal(0, 1)	4.25
	Normal(0, 0.5)	6.75

**Fig 6:**
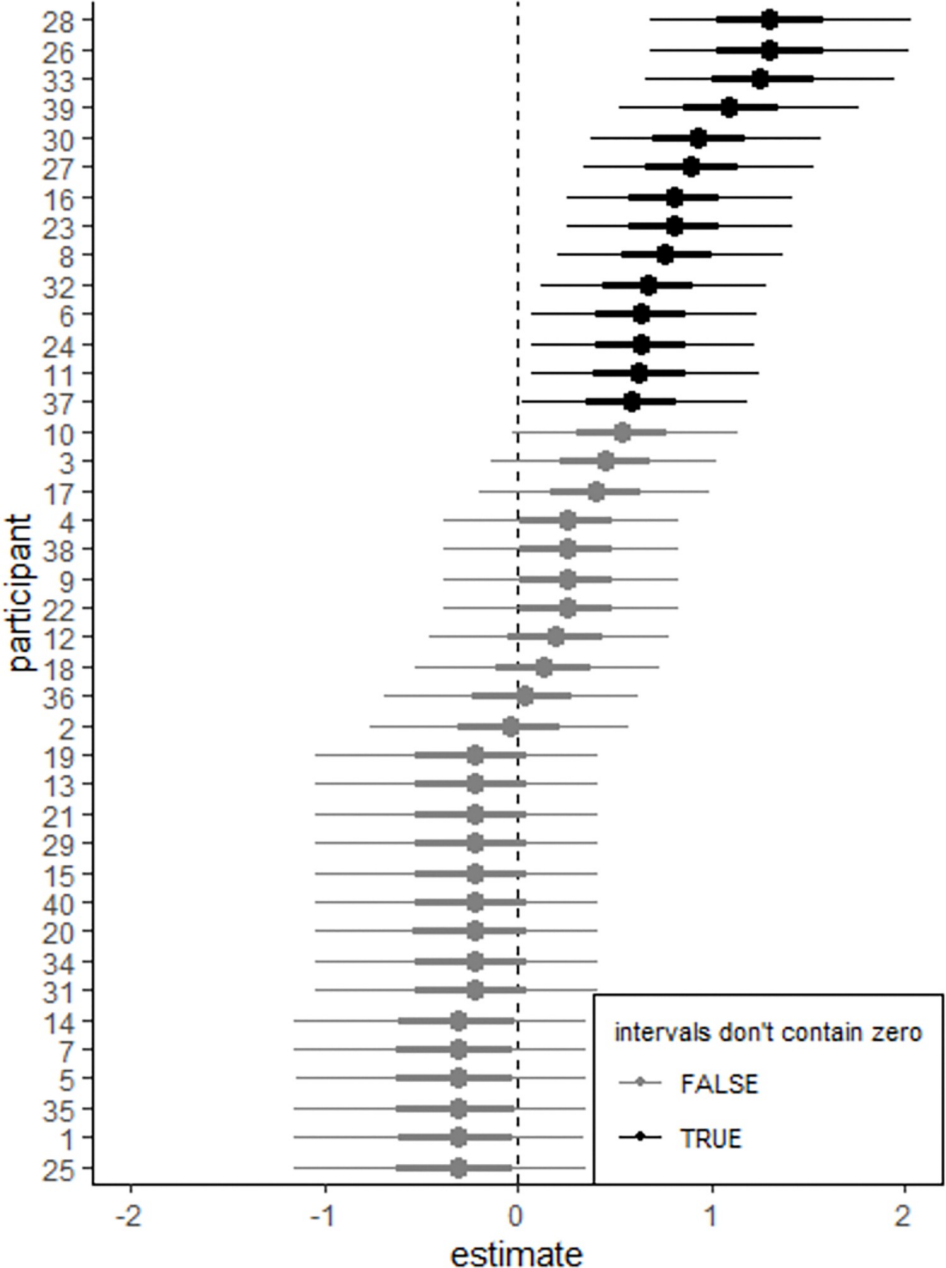
The posterior distribution of the slope for prosody for each participant, averaged over the levels of pair, as visualized using the posterior mean (the black dots) and the percentile-based 95% and 50% posterior intervals (the thin and thick horizontal lines, respectively). Each line in the figure represents a participant with the participant’s ID denoted on the y-axis.

Table 3 also shows that the *BF*_*10*_ evaluating the by-participant varying slopes for prosody ((1+prosody|participant)) exceeded ten across all priors considered, indicating that including this varying slope term was meaningful. Also, Fig 5 shows the 95% CrI for this random slope term, with lower and upper bounds of 0.21 and 0.87, respectively. Both bounds clearly deviate from zero, emphasizing the relevance of including this random slope term in the model. The model showed that the posterior mean of the standard deviation of prosody (sd(prosody)) was 0.51, indicating a relatively large variance of 0.51^2^ = 0.26. Furthermore, Fig 6 shows the posterior distribution of the slope for prosody per participant. It can be observed from Fig 6 that there was a significant variation in the participants’ responses to prosody, with some participants (e.g., participants 26, 28, and 33) responding more strongly to the prosodic manipulations, as evidenced by their 95% CrIs clearly deviating from zero. Conversely, for other participants, the effect of prosody appears less distinct, as their 95% CrIs encompass zero.

In addition to the variance in the slope of prosody for participants, our data also supported the inclusion of the random slope of prosody for pairs. As shown in Table 3, the Bayes factors evaluating by-pair random slopes of prosody ranged from 5 and 11, favoring the model containing the random slopes across the three priors incorporated. Also, the lower and upper bounds of the 95% CrI were clearly away from zero, confirming the necessity of including this random slope term. The model estimates the variance of the random slope of prosody by pair to be 0.42^2^ = 0.17, indicating a non-negligible amount of variability.

## Discussion

The current study investigated the effects of prosody on participants’ interpretation of subjective versus objective causality in English. Specifically, we explored whether the prosodic make-up of the connective word *so* allowed listeners to differentiate between subjective and objective causality. To this end, we conducted a perception experiment online involving a forced-choice discourse completion task. In the task, the participants first listened to audio stimuli announcing real-world events ending with the connective *so*, which was pronounced with either subjective causality prosody or objective causality prosody. Then, they were asked to choose a continuation for each stimulus from two choices shown on the screen, one continuation establishing subjective causality with the stimulus and the other objective causality. Our hypothesis was that if the prosody of the connective adequately signaled subjective versus objective causality, then the participants should be able to correctly anticipate the upcoming causality after hearing the connective. Specifically, we expected them to choose subjective continuations more often after hearing the stimulus presented with subjective causality prosody than after hearing it presented with objective causality prosody and vice versa. Furthermore, given the commonly observed individual differences in perceiving and interpreting prosodic cues, we expected variability among participants in terms of how they interpreted the prosody of the connective *so*. Several interesting findings emerged from our data. Firstly, we found a general bias towards objective continuations regardless of prosody. Secondly, we found that, despite this bias, the prosody of *so* indeed has an effect on the listeners’ interpretation of causality. The connective with subjective prosodic features, that is, a longer duration and a more concave f0 contour, yielded higher odds of choosing subjective continuations. Thirdly, as expected, we found considerable variation among participants in terms of how they reacted to the effect of prosody, with some participants exhibiting a stronger sensitivity to the prosodic features of the connective than others. In addition, we found that the effect of prosody varied across stimuli. In what follows, we discuss these findings in turn and propose directions for future research.

The findings indicate a strong inclination among participants to choose objective continuations, suggesting a preference for constructing objective causality over subjective causality. This bias may stem from the fact that objective causality is easier to construct, which deals with factual situations, as opposed to subjective causality, which is more difficult to construct as it involves the establishment of a meta-representation of other individuals’ mental processes [3, 16, 17, 23]. This presumed disparity in conceptual complexity between subjective and objective causality is supported by evidence from language acquisition and processing, demonstrating that objective causality is acquired earlier by children and requires less time to process than subjective causality [14]. The preference for constructing objective causality may also have been caused by the task design. The continuations, which were adopted from [55], were provided directly to the participants on a computer screen. Although in [55], these items were judged as typical examples of each type of causality by native speakers, the participants in the current study may have been reluctant to accept the judgments or evaluations involved in certain subjective continuations as their own. Consequently, they may have opted instead for the objective continuations. Future studies could explore alternative approaches by inviting participants to generate their own continuations for the audio stimuli they heard, and having annotators to categorize the obtained annotations. However, this procedure would increase the researchers’ workload and could introduce significant variation in participants’ responses.

The most crucial finding of the current study is that although the participants had a baseline inclination towards constructing objective causality, the prosody of the connective *so* had an effect on participants’ choice of continuation. Specifically, when the connective *so* was produced with subjective causality prosody, characterized by a prolonged duration and a more concave f0 contour, the odds of choosing subjective continuations increased by 1.48 times compared to when *so* was produced with objective causality prosody. This finding suggests that the prosody of the connective *so* provides information about the intended type of causality communicated by the speaker. With a prolonged duration and a more concave f0 contour, the connective *so* signals subjectivity, signaling to listeners that the causal relationship between the previous clause and the upcoming clause is established by the speaker’s cognitive reasoning (as opposed to existing in reality). The reason why prosodic features such as prolonged duration and a more concave f0 contour tend to result in higher odds of selecting subjective continuations could be that these prosodic features associate with *subjectivity*. Previous research has found that utterances expressing stances (an umbrella term for subjectivity, evaluation, opinion, and assessment [72]) have longer durations than those stating factual information. Furthermore, a study by [73] has found that the shape of f0 contours vary based on the degree of matter-of-fact conveyed in utterances, with early f0 peaks conveying the highest degree of matter-of-fact and late f0 peaks indicating the speaker’ evaluation. Additionally, a final rise in f0 serves to engage the conversation partner and encourages them to participate in the conversation, whereas a final fall in f0 does not seem to have this effect [74].

This finding improves our understanding of the function of so-called “general causal connectives” such as *so* in processing subjective and objective causality. Previous research has generally found that, in reading comprehension, readers cannot determine the type of causality that the author (or character) intends to convey based on the presence of connective *so* but have to wait for more contextual information to become available, as evidenced by longer reading time for words near the end of sentences [9]. This finding is not surprising because, in written language, general causal connectives are presented only in their lexical forms, which remain unchanged across different types of causality and thus do not distinguish one type of causality from the other. The present study extends this finding by showing that when the connective *so* is presented in listening comprehension, the prosody of the connective can influence the interpretation of causality. By taking into account the prosodic features of the connective, our study shows that listeners are able to derive different interpretations of the nature of causality. Thus, we conclude that spoken instances of the connective *so* can exhibit either subjective or objective characteristics, depending on their prosodic features, rather than being indicative of causality. The prosodic features of these connectives can denote the difference between subjective and objective causality, signaling to the addressees the type of causality the addresser intends to convey. Prosody has been shown to have similar effects in terms of disambiguating different functions or meanings conveyed by polyfunctional words such as *okay* in English and *alors* ‘then/well’ in French. For example, research has found that word-final pitch patterns and word-level intensity can distinguish various functions of the affirmative cue word *okay* [54], and that listeners are able to identify a speaker’s compliance versus reluctance to an interlocutor’s proposal based on the prosodic features of this affirmative cue word [75]. Additionally, a study [53] demonstrated that the prosodic realization of the French discourse marker *alors* ‘then/well’ helps listeners identify the word’s specific function in discourse. Together with these studies, the current study suggests that prosody plays an important role in speech communication by complementing the information provided through the morpho-syntactic level, emphasizing the necessity to take prosody into account when studying the pragmatics of causal connectives as well as other coherence markers. However, it is worth noting that our participants were all between the ages of 25 and 35 and held a bachelor’s degree. While these criteria were necessary for the smooth running of the online experiment, they may limit the generalizability of our findings.

Our data also show that the effects of the prosody of the connective *so* varied across listeners. Individual differences have been found in the interpretation of semantic, syntactic, and discourse information (e.g., [76–79]) and in the perception of prosodic information related to information on these linguistic levels. For example, it has been found that listeners vary in the perception of prosodic cues related to information structure [56], such as focus types [57] and prosodic phrase boundaries [58], and in the interpretation of irony [59] and sarcasm [60]. Several studies have attempted to identify the factors contributing to listener variability. They have found a bouquet of factors that could result in listener variability, including listeners’ cognitive abilities such as working memory [79, 80], fatigue level [81], attention, linguistic experience, autistic traits [56], and also environmental factors such as noises. Among these factors, listeners’ sensitivity to acoustic contrasts might have contributed to the individual variations observed in our data. A person’s sensitivity to acoustic cues plays a role at the early stage of the perception process, allowing a person to discriminate between sounds. After all, if a person cannot perceive acoustic distinctions, they certainly would not be able to utilize the information to establish categorical representations and further apply this category knowledge to decode incoming sounds. Previous research has shown that people who are naturally sensitive to tunes or those who have received musical training have an advantage over others in discriminating sound differences and exhibit enhanced abilities in learning and identifying linguistic timing information [82]. Future research may explore the extent to which musicality accounts for the individual variation in the current study. In addition to listeners’ sensitivity to acoustic cues, given the fact that the current experiment was conducted online, the variability in listener responses observed in our data could also be attributed to the fact that the current experiment was conducted online. While online surveys have several advantages, such as allowing for testing a large number of participants simultaneously and recruiting participants without geographical constraints, they have several limitations. Firstly, the absence of human supervision makes it difficult to know how committed participants were to the task. Secondly, the testing environment cannot be controlled, and factors such as poor internet connection or environmental noises can compromise the sound quality of stimuli. This is particularly problematic for listening experiments that assess subtle acoustic contrasts, such as the subtle contrast between subjective and objective causality prosody tested in the current study. The aforementioned factors might have contributed to the listener variability found in the current study. However, testing participants online was the only option available during the quarantine due to the outbreak of COVID-19. Future studies can reassess listener variability by testing participants in a laboratory setting. Nonetheless, a reviewer has suggested that measures can be taken to enhance data quality when collecting data online. For example, including a trial where participants record a sentence can provide insight into their location and the potential noise level of their environment. Future studies that test the effect of acoustic manipulations online can consider incorporating this measure to improve data quality.

There is yet another possible reason for the listener variability found in the current study. It might be that the prosodic contrasts in our stimuli are not salient enough for every participant to perceive. This likelihood arises because the acoustic contrasts implemented in the current study are based on the findings of [55], in which utterances conveying subjective and objective causality were elicited from the speakers in a controlled laboratory setting, using provided scripts. While we do not think that the findings of [55] deviate from how prosody is actually used in natural conversations to distinguish between subjective and objective causality (for a defense of lab speech, see [83]), it is worth noting that the study design employed in [55] involves a script that is not available in natural conversations and exempts the participants from speech planning. Consequently, it is probable that the prosodic contrasts between subjective and objective causality found in [55] are less dynamic compared to those produced in natural conversations. To evaluate this possibility, follow-up research is needed, implementing prosodic contrasts produced by speakers in a less restrictive conversational environment. Having said that, we acknowledge the alternative possibility that prosodic contrasts found in lab settings might be more prominent than those in natural speech because the experimental conditions of interest are made salient by controlling for many other potentially confounding factors. At the moment, we cannot determine the validity of this explanation, underscoring the need for follow-up research using a less restricted study design.

In addition to individual differences among participants, we also found that the effects of prosody varied between stimuli. For some stimulus pairs, the prosody of the connective did affect the participants’ choice of continuation, while for others, the effect did not seem to be present—the participant preferred objective continuations in both prosodic conditions. We speculate that this variability may be attributed to the semantic content of the provided continuations, where for some participants some subjective continuations may have been more difficult to imagine than the objective ones. Future research is required to investigate this possibility in greater detail. One reviewer has pointed out that, in addition to the semantic content of the provided continuations, the affect conveyed in the provided continuations might act as a confounding factor with the prosody of the causal connective *so*. This means that the preference for subjective continuations may not be solely due to the subjective causality prosody of *so*, but may also be influenced by the type of affect conveyed in the continuations. To examine this possibility, we examined the slope of the effect for each pair of stimuli. We found that the effect of affect on our data was random. Out of the 8 pairs where subjectivity had a positive effect on average, 2 pairs were neutral in terms of affect with the subjective continuation provided, 3 were negative, and 4 were positive. Out of the 7 pairs where subjectivity had a negative effect on average, 2 pairs were neutral in terms of affect with the subjective continuation provided, 3 were negative, and 2 were positive. However, it should be noted that the categorization in terms of affect is rather rough, as it was not the focus of the current study and was not systematically controlled. To investigate the impact of affect on the choice of continuations more accurately, a more nuanced methodology is needed.

## Conclusion

The causal connective *so* in English has long been regarded as a general causal connective that does not distinguish between different types of causality. Through a forced-choice discourse completion task, this study provides evidence that the prosody of the connective *so* allows listeners to interpret the type of the causality the speaker intends to convey. This finding suggests that the prosody of the connective *so* plays a role in conveying subjectivity in causality. This finding thus extends our previous knowledge of the connective *so*, showing that while the lexical form of this connective cannot distinguish between subjective and objective causality, the prosody of this connective can achieve this distinction (at least for some listeners). In light of this, our study underscores the significance of considering prosody when studying the pragmatics of causal connectives.

## Supporting information

S1 Appendix Instructions. (DOCX)

S2 Appendix Items. (DOCX)
