# Supplementary material for: The role of prosody in interpreting causality in English discourse
Source: PLoS One. 2023 Jun 2;18(6):e0286003. doi: 10.1371/journal.pone.0286003 (PMC10237668; doi:10.1371/journal.pone.0286003)
Supplement: S1 Appendix — (DOCX) [file pone.0286003.s001.docx]

# Appendix A Instructions

Welcome to this survey. In this survey you are going to listen to sentences. To be able to perceive the subtle sound variations in the sound bites, please wear headphones or earphones. You are going to listen to sentences introducing events taking place in the real world. After each sentence, you will see two continuations shown in the form of text on the screen. Please select the one you think best continues the sentence you just heard. Please listen to the intonation in the sound clips carefully. You can play the sentences as many times as you want.
