# Supplementary material for: The role of prosody in interpreting causality in English discourse
Source: PLoS One. 2023 Jun 2;18(6):e0286003. doi: 10.1371/journal.pone.0286003 (PMC10237668; doi:10.1371/journal.pone.0286003)
Supplement: S2 Appendix — (DOCX) [file pone.0286003.s002.docx]

# Appendix B Items

Items (in italic) were presented to the participants in audio form. Each target item (1-15) was presented twice, with the connective *so* having objective and subjective prosody. After each stimulus, two continuations (indexed as A and B) appeared on the screen, one forming objective causality with the stimulus and the other subjective causality. In the texts below, for illustration purpose, the objective continuation is always Option A and the subjective continuation Option B; in the experiment, the order of objective and subjective continuation was alternated. Filler items (16-35) were presented once each, after which two continuations were shown on the screen, with Option A being the only reasonable choice and Option B having no semantic relevance (in the experiment, the position of A and B was alternated).

1 *Jim got his nose pierced so*

A: He bled a lot. B: He wants attention.

2 *Alan has a drinking problem so*

A: He goes to AA meetings. B: He has weak self-control.

3 *Tom tripped over his shoelaces so*

A: He fell. B: He is an idiot.

4 *Susan left her wallet on the bus so*

A: She lost her money. B: She is scatterbrained.

5 *Tom went bankrupt so*

A: He moved out of his big house. B: He knew nothing about business.

6 *Jack was always late for work so*

A: He was fired. B: He is not a morning person.

7 *The boat spun around on the current so*

A: Marie fell into the water. B: Marie doesn’t know much about rowing.

8 *Heidi won the first prize at the art festival so*

A: She was happy. B: She is talented.

9 *Hans won the lottery so*

A: He bought a new house. B: He is lucky.

10 *Ian made the school team so*

A: He went to basketball practice. B: He is a talented basketball player.

11 *Joshua played the guitar for hours so*

A: His fingers hurt. B: He has a great passion for music.

12 *Julia has so many books so*

A: She gave some away. B: She loves reading.

13 *The kite is flying so*

A: The girl is laughing. B: The wind is blowing.

14 *Jonny can lift a sofa easily so*

A: He helped his friend move house. B: He is strong.

15 *The boy is playing in the mud so*

A: He gets covered in it. B: He likes mud.

16 *Ben locked himself out of his own house but*

A: He managed to get in. B: Becky wants to take it.

17 *John worked very hard but*

A: He failed his exams. B: The plane landed on time.

18 *Leo went to Paris but*

A: He didn’t visit the Eiffel Tower. B: David found a table.

19 *Bill is a big fan of basketball but*

A: He missed the all-star game yesterday. B: He doesn’t drive it very often.

20 *The new “Star Wars” is nice but*

A: Jessica hasn’t watched it. B: She didn’t go to see the doctor.

21 *Alan was born in China but*

A: He doesn’t speak Mandarin. B: Sam finished it on his own.

22 *Tim is healthy as a horse but*

A: He called in sick today. B: Sam found additional mistakes in it.

23 *Mark tripped over a rock and scratched his knee but*

A: He did not cry. B: They rarely see each other.

24 *Jane and Lisa are neighbors but*

A: They rarely see each other. B: The project didn’t go very well.

25 *Lily’s flight departed later than scheduled but*

A: The plane landed on time. B: He failed his exams.

26 *The math paper was difficult but*

A: Sam finished it on his own. B: He didn’t visit the Eiffel Tower.

27 *Rose liked the dress very much but*

A: She decided not to buy it. B: He missed the all-star game yesterday.

28 *Dylan had planned his project carefully but*

A: The project didn’t go very well. B: He managed to get in.

29 *The café was crowded but*

A: David found a table. B: He was late for the meeting this morning.

30 *The report was already proofread but*

A: Sam found additional mistakes in it. B: Andy had a good time.

31 *The weather was bad but*

A: Andy had a good time. B: He doesn’t speak Mandarin.

32 *Alex didn’t feel very well but*

A: She didn’t go to see the doctor. B: He called in sick today.

33 *Benjamin has a car but*

A: He doesn’t drive it very often. B: Jessica hasn’t watched it.

34 *The job is not well-paid but*

A: Becky wants to take it. B: He did not cry.

35 *Andy is usually very punctual but*

A: He was late for the meeting this morning. B: She decided not to buy it.
